# Green Extracellular Synthesis of Silver Nanoparticles by *Pseudomonas alloputida*, Their Growth and Biofilm-Formation Inhibitory Activities and Synergic Behavior with Three Classical Antibiotics

**DOI:** 10.3390/molecules27217589

**Published:** 2022-11-05

**Authors:** Carlos Pernas-Pleite, Amparo M. Conejo-Martínez, Irma Marín, José P. Abad

**Affiliations:** Department of Molecular Biology, Faculty of Sciences, Biology Building, Autonomous University of Madrid, Cantoblanco, 28049 Madrid, Spain

**Keywords:** antibacterial activity, antibiotics, antimicrobial activity, green synthesis, nanoparticles, *Pseudomonas alloputida*, ROS, silver, synergy

## Abstract

Bacterial resistance to antibiotics is on the rise and hinders the fight against bacterial infections, which are expected to cause millions of deaths by 2050. New antibiotics are difficult to find, so alternatives are needed. One could be metal-based drugs, such as silver nanoparticles (AgNPs). In general, chemical methods for AgNPs’ production are potentially toxic, and the physical ones expensive, while green approaches are not. In this paper, we present the green synthesis of AgNPs using two *Pseudomonas alloputida* B003 UAM culture broths, sampled from their exponential and stationary growth phases. AgNPs were physicochemically characterized by transmission electron microscopy (TEM), total reflection X-ray fluorescence (TXRF), infrared spectroscopy (FTIR), dynamic light scattering (DLS), and X-ray diffraction (XRD), showing differential characteristics depending on the synthesis method used. Antibacterial activity was tested in three assays, and we compared the growth and biofilm-formation inhibition of six test bacteria: *Bacillus subtilis*, *Escherichia coli*, *Klebsiella pneumoniae*, *Pseudomonas aeruginosa*, *Staphylococcus aureus*, and *Staphylococcus epidermidis*. We also monitored nanoparticles’ synergic behavior through the growth inhibition of *E. coli* and *S. aureus* by three classical antibiotics: ampicillin, nalidixic acid, and streptomycin. The results indicate that very good AgNP activity was obtained with particularly low MICs for the three tested strains of *P. aeruginosa*. A good synergistic effect on streptomycin activity was observed for all the nanoparticles. For ampicillin, a synergic effect was detected only against *S. aureus*. ROS production was found to be related to the AgNPs’ antibacterial activity.

## 1. Introduction

The antibiotic resistance of pathogenic bacteria is one of the most pressing problems that humanity will have to deal with in the coming years [1], and is already considered a silent pandemic [2] that will cause an estimated 10 million deaths per year by 2050 [3]. Therefore, action should be taken to address this situation as soon as possible. Since not many new antibiotics are coming out, other alternatives should be studied. One of the most promising ones is the use of metal-based drugs, particularly metallic nanoparticles [4], even though some possible drawbacks also need to be studied [5]. Among metallic nanoparticles, ones based on silver are the most studied. Silver has long been known to be a good antibacterial agent in the form of monovalent silver salts, but its use in the form of nanoparticles seems to be more convenient and it has been studied extensively [6,7].

A huge number of methods have been described for the synthesis of silver nanoparticles (AgNPs), based on chemical, physical, or biological approaches. The use of chemical or physical methods usually produces a more regular shape and size of individual nanoparticles than biological methods [8,9,10]. However, for use as antimicrobial drugs, that may be irrelevant in most cases. The biological methods employ extracts or culture broths containing reducing and protecting agents generating silver nanomaterials, whose activity depends on the method and the biological material used [11,12,13,14,15,16]. Since no toxic chemicals are used and the biological material is usually not expensive nor requires sophisticated equipment, biological methods are considered more convenient and greener. Most of these employ extracts of different plant parts such as seeds, leaves, flowers, etc. [11,16,17], but animal blood serum [18], invertebrates [19], algae [20], lichens [21], fungi [22], and bacteria [11,23,24] have been used as well. Different organisms may provide different physicochemical characteristics and biological properties to the nanoparticles.

Microorganisms are the second more frequently used source for the biogenic synthesis of AgNPs, especially bacteria. In these cases, intracellular and extracellular methods have been described that use extracts or culture broths of a wide diversity of bacteria, such as *Paenarthrobacter nicotinovorans* [24], *Klebsiella pneumoniae* [25], various *Pseudomonas* species [26,27], actinobacteria [28], psychrophilic bacteria [29], and even an endophytic *Bacillus cereus* [30], among many others. Using various biological materials increases the possibility of producing AgNPs with different properties. Though plants have most often been used to produce AgNPs, microorganisms are much more phylogenetically diverse and would require less physical space and growth time than plants for producing biological material in better controlled and reproducible growth conditions. So, research on new species of bacteria able to produce AgNPs could be a good approach to obtain ones with different properties, which would then allow us to select the most appropriate ones for each application. Additionally, using different media [31,32] for culturing the same bacteria or cultures from different growth phases may provide additional possibilities for generating AgNPs with different properties.

One of the main problems with using AgNPs as antimicrobial agents is the possible cytotoxicity. However, diverse studies have described different levels of toxicity of several AgNPs produced by different methods [33], from no toxicity [34,35] to high toxicity [36]. Thus, new approaches for AgNP synthesis, particularly when employing diverse biological materials, would eventually produce AgNPs that could be used in clinic. In this sense, if AgNPs show antimicrobial activity at lower concentrations than those in which they produce significant toxicity, a high possibility exists that they could be used as antibacterial agents, as several authors have claimed [33,37,38].

Another possibility is for AgNPs to be used for fighting infections in combination with classical antibiotics. This would allow us, if synergy occurs, to use lower concentrations of antibiotics and AgNPs than when they are used alone [39,40]. Moreover, recent studies have shown that AgNPs can be used to recover the use of certain classical antibiotics not regularly used because of their toxic effects or resistance [41,42].

The objectives of this study were to synthesize new types of AgNPs by a green method using *Pseudomonas alloputida*, a species never before used for AgNP production, and to determine their structural characteristics as well as their properties as antibacterial agents. As stated above, biological diversity could be helpful for producing new and different AgNPs with distinct properties. Thus, we wanted to determine if the same bacterium could also be employed to produce several types of AgNPs by using broths sampled in two growth phases, exponential (bacteria actively growing) and stationary (arrested growth), and using two media, one with and another without NaCl. Structural differences between the various AgNPs produced, as well as their antibacterial activity, would tell us if the AgNPs are different, and what their potential to be used as antibacterial agents is. Among the possible antibacterial tests, we were interested in performing quantitative determinations of the growth and biofilm-formation inhibition of various Gram-positive and Gram-negative bacteria. One of the most interesting putative applications of AgNPs is as helpers of classic antibiotics in a synergistic way, and we also wanted to evaluate this possibility for our AgNPs. Finally, we wanted to test if the AgNPs produce reactive species of oxygen (ROS) and their correlation with the antibacterial activity parameters to advance the study of their mechanism of action.

## 2. Results and Discussion

The work presented here addresses the production of AgNPs by biological means, to be used as antimicrobial agents. We used a *Pseudomonas* strain that had been isolated from the Tinto River estuary (Huelva, Spain) in a previous study on bacterial antibiotic resistance in this environment [43]. Before using this isolate, it was phylogenetically identified as follows.

### 2.1. Phylogenetic Ascription of the Bacterial Strain Used in AgNP Synthesis

**Figure 1:**
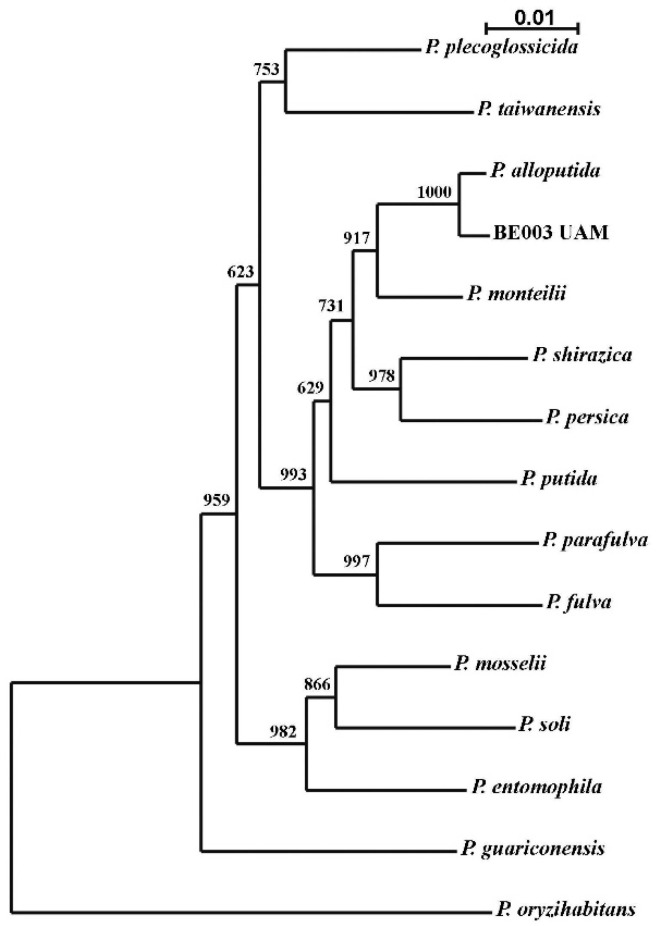
Neighbor-joining phylogenetic tree for assignment of the isolate B003 UAM to *Pseudomonas alloputida* species. Sequences from the type strains of each species were used. Numbers are bootstrap values of the corresponding branching points. The bar indicates the scale of phylogenetic distances of the branches.

Identification of the species to which this isolate belongs was performed based on a two-locus analysis with concatenated sequences of 16S rDNA and the *rpo*D gene. A neighbor-joining phylogenetic tree showed maximum proximity to the type-strain of *P. alloputida* (Figure 1). This was corroborated by the percentages of sequence similarity for both genes. A sequence of 1383 nucleotides of the 16S rDNA and one of 688 of the *rpo*D gene were used, obtaining 99.85% and 98.20% similarity, respectively. Even though the 16S rDNA is not considered discriminative enough for *Pseudomonas* species, the similarity of the analyzed sequence of *rpo*D gene was over the minimum required to identify our isolate as *P. alloputida* [44,45]. *Pseudomonas* is an interesting genus because of its biotechnological potential [46], and applications for *P. alloputida* have been described [47,48]. As far as we know, this species has not previously been used for AgNP synthesis.

### 2.2. Pseudomonas alloputida B003 UAM Cultures and Growth Curves

The growth of the bacterium was tested in a regular nutritive medium or a NaCl-deprived one, as the presence of this salt is known to affect AgNP synthesis [29,49] and could induce the production of different types of AgNPs. Growth curves showed that the bacterium grows faster in a regular medium (Figure S1, Supplementary Materials), indicating different behavior depending on the medium used, which might affect the broth composition and properties. As another variable to produce different AgNPs, cultures were sampled at exponential and stationary phases from each medium. We have not found reports of using bacterial broths from different growth phases for producing different types of AgNPs, but we considered this a good way to obtain different biological materials since, in the exponential phase, bacteria are in active growth, producing many different compounds, while in the stationary phase only maintenance processes are taking place.

### 2.3. AgNPs Biosynthesis

**Figure 2:**
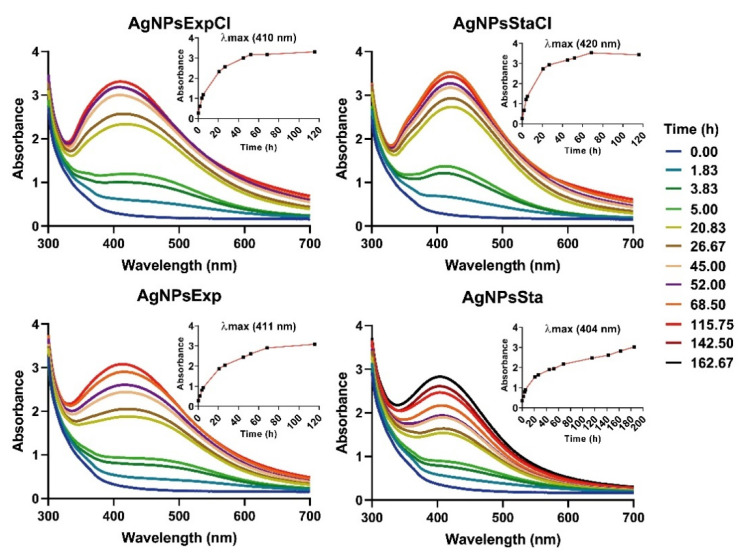
UV-Vis spectra of AgNPs over time during their synthesis. The inset figures in the upper right corner of each panel show the kinetics of synthesis from the increase in absorbance at the corresponding λ_max_.

AgNPs syntheses were followed spectrophotometrically (Figure 2). The highest absorbances were observed at about 40–60 h of synthesis for the AgNPsExpCl and AgNPsStaCl (see Section 3.3 for the naming of the AgNPs), 60–80 h for AgNPsExp, and 180–200 h for the AgNPsSta. With longer reaction times, a decrease in the absorbance at λ_max_ and an increase at higher wavelengths (>550 nm) was observed, indicating rearrangement or aggregation of the AgNPs (this can be seen in Figure 2 for the longer reaction times, mainly for the AgNPs from a regular medium). For all our structural and antibacterial activity determinations, AgNPs were collected from the synthesis reactions before any sign of aggregation occurred. After the washing steps, spectra were registered to determine the integrity of the AgNPs. We observed no significant changes (Figure S2), which indicated the conservation of the structure of all AgNPs during this process.

The results showed differences in the speed of synthesis depending on the NaCl content of the broths used and the growth phases from which the broths were taken. Synthesis of AgNPs using media with NaCl was faster with no dependence on the growth phase. However, syntheses were slower when a NaCl-deprived medium was used and, in this case, different speeds were observed depending on the growth phase. The slowest were those from the stationary one. The presence of NaCl in the broth seems to facilitate nanosilver production, but we cannot determine if this effect is due to NaCl itself or the presence of different compounds in the broths, with or without NaCl, as the different growth of the bacterium in the two media may indicate. As some authors have suggested [29,49], the presence of Cl^−^ could lead to the formation of AgCl crystals. Due to the low solubility of this compound, it may precipitate forming crystals that could have a nucleation effect on the synthesis of AgNPs, thus accelerating this process. Since the XRD analyses, which will be described later, have shown that AgNPs synthesized in the presence of NaCl contain AgCl crystals, we can guess that these were nucleating the AgNPs. In the literature, a wide range of synthesis rates for silver nanoparticles has been reported depending on the biological material used or the synthesis method and conditions, such as pH [50], temperature [29,50], or NaCl presence [29,49]. In previous work by our group [29], the presence of NaCl did not speed up AgNP synthesis; the contrary was true. This may indicate that the effect of NaCl could also be related to the bacteria used in the AgNP synthesis. The time needed for the green synthesis of AgNPs has been reported as rapid—a few minutes, as described by Mokhtari et al. (2009) [25] to hours [26]—or longer, taking days [28,36] or even several weeks, as, for instance, was described by Javani et al. (2015) [29]. In our case, the maximum production was achieved in an intermediate time.

To check the reproducibility of the synthesis, three batches of AgNPsSta and AgNPsStaCl were prepared as models and their kinetics compared (Figure S3), also checking the stability of the nanoparticles at 4 °C over the two weeks after synthesis and washing. Structural stability was evaluated by the UV-Vis spectra, and the conservation of antimicrobial activity by comparison of the minimal inhibitory and bactericidal concentrations (MIC and MBC) (Figure S4). The results indicated the good reproducibility of the kinetics of synthesis, stability of the spectra, and conservation of antibacterial activity. These results allow us to use each AgNP batch for at least two weeks after synthesis, when conserved at 4 °C. Nevertheless, each batch was kept at 4 °C and used for no more than one week after synthesis; spectra were rechecked just before use in each physicochemical analysis or antimicrobial activity test. The preparation of several batches of each type of AgNPs was needed during this study, and the kinetics of each were routinely checked to ensure repeatability. Once the reproducibility of the synthesis and spectra were checked, physicochemical and antibacterial activity characterization of the AgNPs was performed.

As a test to determine if the broths used once for AgNP generation still have the capability to synthesize AgNPs without deleterious consequences on the AgNPs produced, the broth used to prepare the AgNPsStaCl was reused for a new synthesis. This was performed two consecutive times without apparent changes in the kinetics of synthesis and obtained nanoparticles with the same UV-Vis spectra (Figure S5). This allows the synthesis of several batches from the same broth stock, decreasing the production costs. As far as we know, this is the first time that reusing biological material for producing consecutive batches of AgNPs has been reported.

### 2.4. Characterization of Biosynthesized AgNPs

#### 2.4.1. UV-Visible Spectrophotometry

AgNPs showed typical UV-Vis spectra, as can be seen in Figure 2, with some differences in λ_max_ for nanoparticles synthesized from the stationary phase of regular medium with the largest value (420 nm). A lower, but similar, λ_max_ value was found for the AgNPs prepared with exponential broths (410–411 nm), while for those from the stationary phase a clear difference was observed, with the lowest value for AgNPsSta (404 nm). These values correlate with the AgNP sizes observed by TEM (see below), which agrees with the clear relationship between the λ_max_ and the AgNP size claimed by other authors [51].

#### 2.4.2. Elemental Composition of AgNPs by Total Reflection X-ray Fluorescence (TXRF)

**Figure 3:**
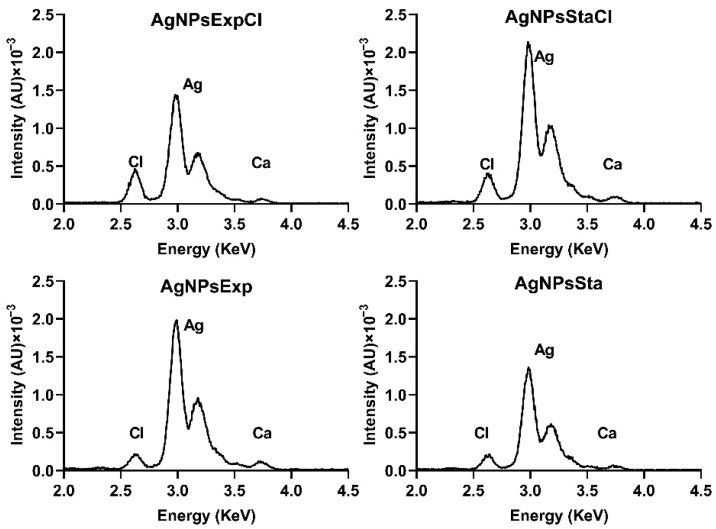
TXRF analysis of elemental composition of AgNPs around the silver peaks. AU: arbitrary units.

TXRF was used to determine the elemental composition of the AgNPs and the silver concentration of nanoparticle suspensions. AgNPs were mainly made of silver, with insignificant amounts of some other elements, except for Cl. Silver appeared as two adjacent peaks in the 3 KeV region, and close to them was a small one at 2.6 KeV corresponding to chlorine (Figure 3). AgNPs prepared from regular nutritive broths contained a higher proportion of Cl.

The chlorine present in the AgNPs prepared in a medium without added NaCl could come from the residual chloride salts in the extracts used in the preparation of the culture media. The low, but significant, amounts of Cl in the AgNPs prepared with a NaCl-containing medium come from the Cl provided by this salt, as found in other reports [52,53].

#### 2.4.3. Crystallinity by Powder X-ray Diffraction (XRD)

**Figure 4:**
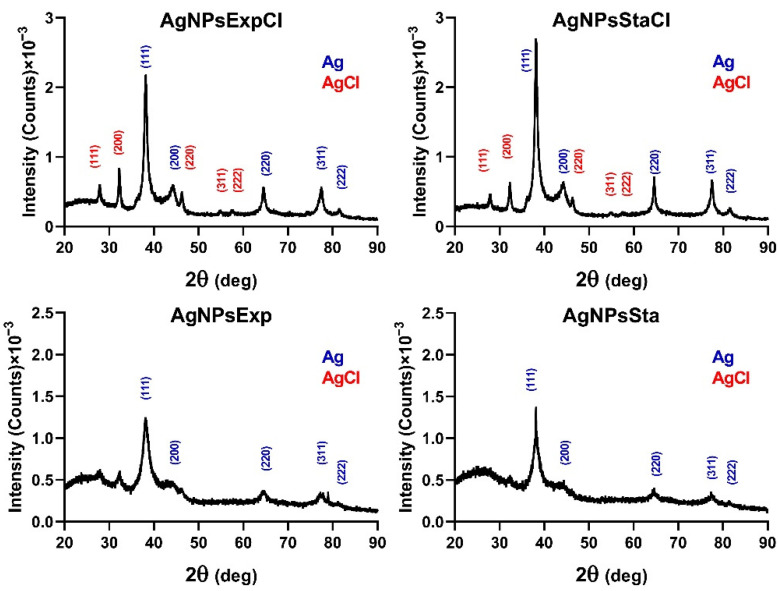
Nanoparticles’ crystallinity determination. XRD patterns of the AgNPs with the indicated planes of Ag^0^ (blue) and AgCl (red) crystals.

The results of XRD for all nanoparticles tested showed peaks in 2θ values around 38.22, 44.01, 64.58, 77.53, and 81.33 degrees, corresponding to the (111), (200), (229), (311), and (222) planes, respectively, of the face-centered cubic structure of metallic silver crystals (Joint Committee on Powder Diffraction Standards (JCPDS) file 04-0783) (Figure 4). Those nanoparticles prepared with broths from a regular nutritive medium showed additional peaks at 27.89, 32.25, 46.26, 54.86, and 57.52 degrees, corresponding to the same planes, associated with a face-centered cubic structure of AgCl crystals (JCPDS file 31-1238), indicating a detectable amount of this compound in these nanoparticles.

XRD analysis of the AgNPs showed some differences between AgNPs produced in regular or NaCl-deprived media. Those produced in a medium with NaCl contained detectable amounts of AgCl crystals, as their specific peaks were observed. AgNPs synthesized in a medium without NaCl only showed detectable peaks corresponding to Ag^0^ crystals, even though a smaller amount of chlorine was also detected in their TXRF analyses. Several articles have described the detection of AgCl in the XRDs of their silver nanoparticles [52,53], attributing it to the presence of NaCl in the broths used in their synthesis [52]. The presence of this quite water-insoluble material in the AgNPs could affect their activity because of the difficulty of releasing silver ions and may be one of the factors responsible for the lower antibacterial activity showed by AgNPsExtCl and AgNPsStaCl. However, the nanoparticles of AgCl (AgClNPs) described in the literature still show antibacterial activity [52,53]. It is quite curious that the synthesis of AgNPs in media containing NaCl can produce materials in which the AgNPs are predominant versus the AgClNPs (this paper), or in which AgClNPs predominate [52,53]. Since the same concentration of NaCl is present in these cases, this cannot be the determining factor in the predominant chemical form, Ag^0^ or AgCl, in which silver is found in these nanomaterials. Other factors such as the composition of reducing and protecting agents in the broths, or the methodological conditions, should have some influence on this. Perhaps the concentrations of reducing and capping agents in the biological material used for synthesis could be involved in this phenomenon. If high concentrations are present, they could, totally or partially, prevent the formation of AgCl crystals, nucleating and capping the Ag^0^ cores. However, if the concentrations are low, the formation of AgCl crystals before most of the silver is reduced and cores capped could occur.

#### 2.4.4. AgNPs’ Core Shape and Size, as Determined by Transmission Electron Microscopy (TEM)

**Figure 5:**
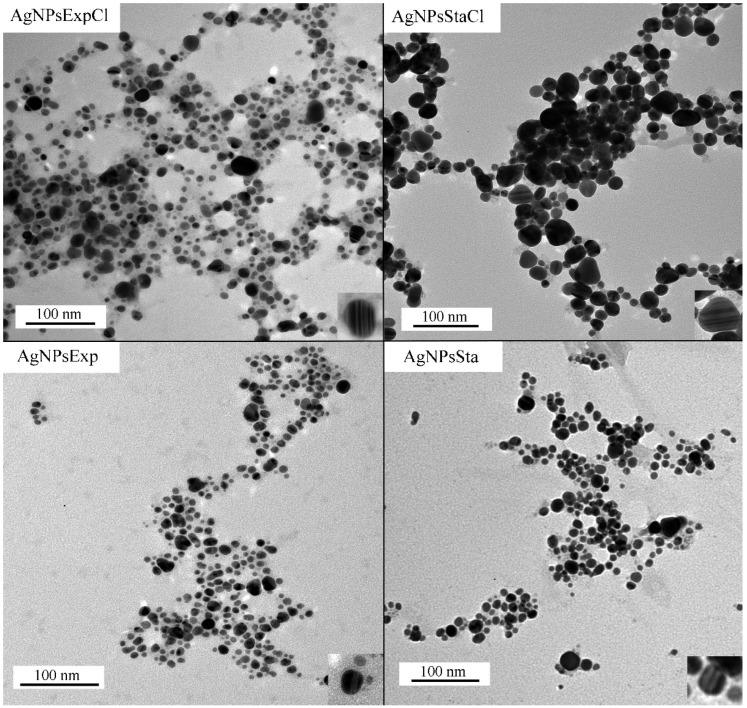
TEM representative images of AgNPs. Lower-right corner insets in each panel show an amplified image of a nanoparticle with internal parallel patterns.

TEM images show the electrodense core of the AgNPs, from which their shape and size can be determined (Figure 5). The four types of AgNPs presented mostly quasi-spherical shapes, but other shapes were also found in a few nanoparticles. In some particles, higher electron density structures arranged in parallel lines were observed (examples are shown in the inset images in the lower-right corners of the panels in Figure 5).

**Figure 6:**
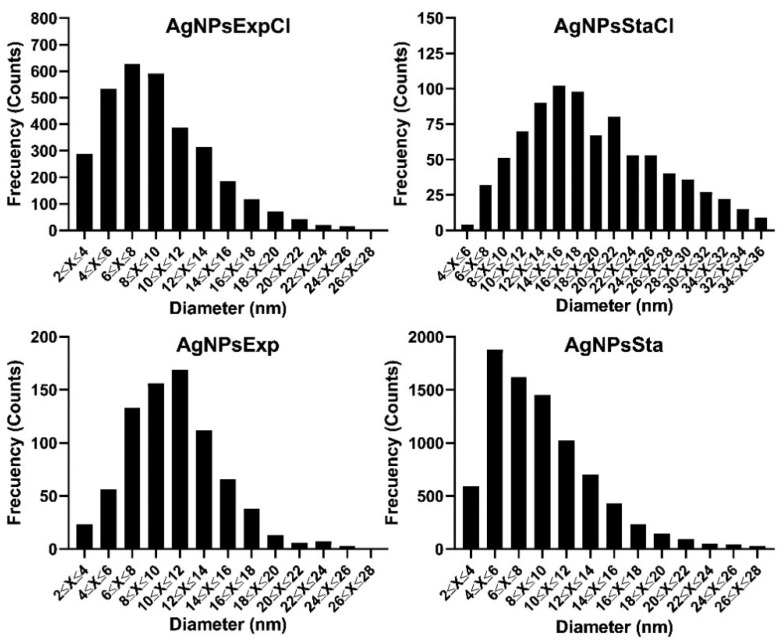
Distribution of AgNPs’ core diameters.

From the analysis of several TEM images for each type of AgNPs, the average diameter of the AgNPs’ cores and the polydispersity of their sizes were determined (Table 1). Average sizes were quite similar for all the AgNPs, except for AgNPsStaCl, which was almost double the size of the others. Size distributions were also assessed (Figure 6), allowing us to determine the AgNPs-cores’ size polydispersity (Table 1).

The polydispersity index (PDI) values are quite similar for the various types of AgNPs, except for AgNPsSta, which is higher. In most cases, they correspond to moderate polydisperse suspensions following the nomenclature described in [54].

Recent reviews on the green synthesis of AgNPs show that a wide range of nanoparticle sizes and polydispersity can be found in the literature [9,12,13], with spherical shape being the most frequent. For instance, in the articles published from 2015 to 2021 that were summarized by Vishwanath and Negi (2021) [9], AgNPs with sizes from 2 to 200 nm, some over the limit of the nanoparticle scale, were reported. In this range, we could say that our AgNPs are rather small. Polydispersity of the AgNPs’ sizes is also quite variable between reports, with monodispersity or low dispersity being scarce.

#### 2.4.5. Zeta-Potential and Hydrodynamic Diameter of AgNPs by Dynamic Light Scattering (DLS)

All the AgNPs presented a negative Z-potential, with higher values for nanoparticles synthesized using exponential phase broths, around −31 to −35 mV, than for those from stationary phases with values around −20 to −23.5 mV (Table 1). These results suggest the moderate to high stability of these materials, especially for those from exponential phase broths, assuming the generally considered relationship between Z-potential absolute values and the stability of colloids. However, such a direct relationship may not exist in many cases, as reported by Bhattacharjee (2017) [55]. No significant differences were observed based on the presence or absence of NaCl in the broths. Our test for the stability of AgNPs, prepared with stationary phase broths and based on UV-Vis spectra and antibacterial activity determination, also indicated good stability in water at 4 °C for at least two weeks, as has been indicated above.

Some authors have claimed a relationship between Z-potential and the antimicrobial activity of nanoparticles [56], suggesting that more negative values would correspond to AgNPs exhibiting reduced activity, because the repulsion of negatively charged bacterial envelopes would make it difficult to approach them. This is not the case for our AgNPs, as will be discussed below.

The average hydrodynamic diameters of the AgNPs (Table 1) were significantly different for the four types of AgNPs, with the smallest being that of AgNPsExp and the largest that of AgNPsSta. More similar were those of the AgNPs prepared with a regular nutritive medium. The size polydispersities were similar, around 0.30–0.34, indicating a moderate level for most AgNPs; the exception was AgNPsExp with a value of 0.45, slightly above the limit of moderate dispersity. Hydrodynamic diameters, as measured by DLS, show larger values than the core sizes, as can be expected. However, no correlation exists between these two sizes. We could speculate that media from the stationary phase may have higher concentration of compounds than those from the exponential phase, or different or additional ones, that may interact, producing a thicker corona. The presence of NaCl might regulate the addition of compounds on the AgNP cores, homogenizing the hydrodynamic size on cores of different size. Moreover, in [57], the authors found that NaCl can affect the hydrodynamic size, which may help to explain the findings of this study.

#### 2.4.6. Corona Composition by Fourier Transform Infrared Spectroscopy (FTIR)

**Figure 7:**
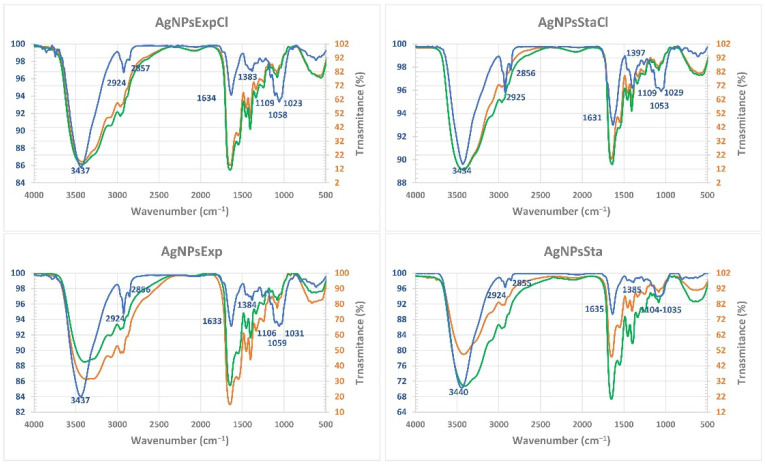
Characterization of the corona components of AgNPs and broths. AgNPs’ spectra are shown in blue; the broths before synthesis are in orange and after synthesis in green. The wavenumber of the principal bands of AgNPs are indicated.

FTIR spectra showed almost no differences between the broths before and after the AgNP synthesis, with or without NaCl, but some differences could be detected among the various AgNPs and with the corresponding broths (Figure 7). In general, the infrared bands of the nanoparticles were thinner than those of the broths, indicating a simpler composition of the AgNPs’ coronas. Moreover, differences in the fingerprint region can be observed between the various AgNPs, indicating their differential composition. In this region, a particular pattern is observed for each AgNP, but similarities can be observed between those coming from the same phase, from cultures with or without NaCl. Thus, the bands at around 1630 cm^−1^ are more intense that the triplet bands around 1060 cm^−1^ in the case of stationary phase AgNPs, and of similar intensity to those from the exponential one. The spectra of the two AgNPs from the stationary phase show differences in the bands around 1380–1400 cm^−1^ between those produced in a medium with NaCl or without it, with a higher intensity in the AgNPs from a medium with NaCl. Differences in this region between the AgNPs from exponential phase cultures are much less evident. These observations indicate a different composition among the four types of nanoparticles’ coronas, with those obtained from the same growth phase being more similar, and less significant differences related to the presence of NaCl. The main bands of the AgNPs’ IR spectra would correspond to vibrations of protein bonds (3430–3440 cm^−1^ and 1631–1635 cm^−1^ regions) and carbohydrates (1023–1106 cm^−1^ region), with minor contributions of other biomolecules. We used the barcode signals reported in [58] to ascribe the main FTIR bands to each type of biomolecule.

These results suggest that, although different conditions of synthesis are used, AgNPs tend to be surrounded by the same kind of organic compounds. Probably, differences in corona components exist, in terms of the proportion of each type of a particular compound, producing properties that may diversify its functionality. The absence of easily detectable differences in the FTIR spectra of the broth before and after synthesis tells us that most of the components detected were in high amounts, but only a small portion of them was used and attached to the silver core of the AgNPs. This agrees with the ability of the used broth to produce nanoparticles in at least two successive batches, as indicated above. The differences between the broth and AgNPs’ spectra indicate a selection during their synthesis of the components of the broth that remain on the corona. In the FTIR studies of other green synthesized AgNPs, different types of compounds have been reported; thus, for instance, Kumar and Mamidyala (2011) [26] determined the presence of proteins and lipids in the AgNPs produced by a supernatant of *Pseudomonas aeruginosa*, or in other cases, different spectra were reported [27,28,30], in most cases detecting the presence of proteins and other not well identified compounds. These different results would be related to the composition of the biological materials used in the biosynthesis and reinforce the possibility of obtaining AgNPs with different composition and properties based on biological diversity.

### 2.5. Antibacterial and Antibiofilm Activity of AgNPs

We evaluated each type of AgNPs for the growth inhibition of three Gram-negative bacteria and three Gram-positive ones by the microdilution method. The antibacterial activity parameters minimal inhibitory concentration (MIC), minimal bactericidal concentration (MBC), inhibitory concentration of 50% growth (IC_50_), and inhibitory concentration of 50% biofilm formation (ICb_50_) were also calculated (Table 2). Overall, the MIC values ranged between 0.24 and 9.68 µg of Ag/mL, depending on the AgNP type and bacterium tested. All the AgNPs showed a good activity (MIC < 10 µg of Ag/mL), with very low values for the four parameters against all bacteria. A recent review on the antibacterial activity of AgNPs described, for most of them, higher MICs values than those determined for our AgNPs [59]. For none of our AgNPs do the values of the antimicrobial activity parameters differentiate between Gram-negative and Gram-positive bacteria, but differences were observed among individual species.

The highest MICs were for AgNPsStaCl, which ranged between 2.42 µg of Ag/mL for *P. aeruginosa* to 9.68 for *K. pneumoniae* and *S. aureus*, while, except for *S. aureus*, the most active was AgNPsExp, with values from 0.24 µg of Ag/mL for *P. aeruginosa* to 1.94 for *S. aureus*. Similar results were obtained for MBC and for IC_50_ and ICb_50_. The most susceptible bacterium to all AgNPs was *P. aeruginosa*, with MICs depending on AgNP type from 0.24 to 2.42 µg of Ag/mL and lower for *S. aureus* at 1.44–9.68 µg of Ag/mL. The results obtained indicated a higher activity for all the synthesized AgNPs against *P. aeruginosa* CECT 108, so we decided to include in the study two more strains of *P. aeruginosa*, from different origins, strains PA01 and PA14, to assess whether the lower values of the inhibitory concentrations were due to the species or the strain used. We chose AgNPsSta for this experiment because they have intermediate efficacy. The three strains showed similarly high susceptibility, suggesting that these AgNPs are especially active against this species. Some AgNPs have also been described as highly effective against *Pseudomonas*; for instance, AgNPs producing 99.9% of bacteria death at 5 µg/mL have been reported [60], and a 1 µg/mL MIC on *P. aeruginosa* ATCC 10145 has been described for other AgNPs, which required 4 µg/mL for inhibiting biofilm production [61]. However, very different levels of susceptibility have been reported for different strains, depending on the AgNPs used, with MICs from 1 µg/mL to 1 mg/mL [62].

In general, MIC and MBC values of the AgNPs were the same or MBC ≤ 4 × MIC, revealing a bactericidal mode of action in all bacteria evaluated for most of the AgNPs, as suggested in [63].

Physicochemical characteristics such as the shape, size, and Z-potential of AgNPs have been correlated with their antimicrobial activity. Thus, several reports have shown the effect of AgNPs shape on antimicrobial activity, sometimes with contradictory results. In some cases, a cubic shape seems to be more active than a spherical one [64]; in others, a triangular shape seems to be the most active [65]; in others still, the spherical shape has been found to be more active than triangular or others [66]. All our AgNPs were mostly spherical; moreover, good antimicrobial activity was found. Considering the AgNPs’ sizes, the smallest have been associated with the best antimicrobial activity, due to their greater surface area [51,59,67,68]. In this report, the less active of our AgNPs had the largest core, while a range of higher activities was observed for the other three with similarly small sizes. This indicates a certain correlation between core size and antibacterial activity. However, the activities of the smaller AgNPs show larger differences than the sizes themselves. Probably, differences in the corona composition also have an effect on the observed antibacterial activity, as suggested by other reports in which a correlation of size and activity has not been found [29,69]. With respect to the Z-potential, more negatively charged particles have been suggested to be less active because of the eventual repulsion of the bacterial cell envelopes, also negatively charged [56]. However, in our case, the most negatively charged AgNPs are the AgNPsExpCl and the AgNPsExp, which, according to their MICs, are not less active; moreover, the AgNPsExp are the most active against most of the tested bacteria. The less charged ones are the AgNPsStaCl, but they are also less active. The effect of the Z-potential on AgNP activity is probably modulated by other factors in a still unknown way. For instance, the sign of the Z-potential can even change depending on different factors such as pH [55]. Probably, for AgNPs that are chemically or physically produced, with low-complexity coronas, this correlation may be appropriate, but because of the high complexity of the green synthesized AgNPs, other factors may be acting to prevent such correlation.

The formation of biofilms by bacteria is usually a form of protection. It is known that the difficult penetration of substances in these structures is a way to diminish the action of antibacterial drugs [70,71]. Due to their sizes and surface properties, AgNPs have been evaluated as antibiofilm agents [72,73]. We have determined this activity of the AgNPs reported here and express it as ICb_50_ values (Table 2). These are all low, indicating the good ability of these materials to inhibit biofilm formation. In general, the comparison of ICb_50_ with IC_50_ shows the higher values of the former, indicating that these AgNPs are less active against biofilm formation than at inhibiting the growth of planktonic cells, but still showed good antibiofilm activity (ICb_50_ < 6.20 µg of Ag/mL). In other studies, some AgNPs have been shown to prevent the formation of biofilms by various bacteria; thus, for instance, it has been described that some commercial AgNPs were able to produce around 50% inhibition of biofilm formation at concentrations about 50 ppm for *E. coli* and a little higher for *S. aureus*, when MIC values were 6.25 and 12.5 ppm [73], respectively, showing quite a lower efficiency than our AgNPs.

### 2.6. Synergy of AgNPs with Classic Antibiotics by the Checkerboard Assay

Several studies have shown the ability of AgNPs to act synergistically with other drugs, such as antibiotics, in the inhibition of bacterial growth [74]. Therefore, we evaluated the possible synergy between our AgNPs and three classic antibiotics by the checkerboard assay [75,76]. The results of this analysis were analyzed based on the fractional inhibitory concentration index (FICI) [75,76,77] and the modulatory factor (MF) [40], for each antibiotic and AgNP combination (Table 3). A strong synergistic effect was observed between any of the AgNPs and streptomycin against *E. coli* and *S. aureus* (FICI ≤ 0.5; MF = 16–32). Synergy was also detected with ampicillin, but only against *S. aureus* (FICI ≤ 0.5; MF = 2–4). Moreover, additive activity (0.5 < FICI ≤ 1; MF = 1–2) was shown by AgNPsExp, AgNPsExpCl, or AgNPsStaCl in combination with nalidixic acid against *E. coli*. Indifferent effects were observed for all AgNPs with ampicillin against *E. coli*, as well as nalidixic acid against *S. aureus* and for AgNPsSta against *E. coli* (1 < FICI ≤ 2; MF = 1). No antagonistic effect (FICI ≥ 4) was detected. Using a stricter interpretation of FICI [75], a synergistic effect can be considered for all AgNPs with streptomycin against both bacteria and with ampicillin against *S. aureus*. The synergy observed by other authors was dependent on the AgNPs used, bacterial species tested, strains, and antibiotic. In previous studies recently reviewed by Ribeiro et al. [74], the most tested antibiotic was ampicillin, followed by streptomycin, while nalidixic acid was scarcely evaluated, and *E. coli* and *S. aureus* were the most frequently used bacteria. Depending on the study and the AgNPs used, positive or no synergy with ampicillin was reported; sometimes synergy of the same AgNPs was observed against either *E. coli* or *S. aureus*, both, or neither. For streptomycin, in most cases *E. coli* and *S. aureus* were synergistically inhibited by AgNPs, and only in a few cases was synergy only observed against *E. coli*. This illustrates the different behavior of various AgNPs depending on the method of synthesis and their physicochemical characteristics, though a correlation between the synergistic effects and any of the AgNP characteristics has not been described. The combined use of AgNPs and classic antibiotics may help to decrease the concentrations needed and perhaps the cytotoxic effects, as shown in [78]. This can also be useful for the recovering of antibiotics not used because of high levels of pathogen resistance, toxic effects on bacterial hosts, and perhaps the impossibility of reaching sufficient concentrations in the host’s fluids or tissues for effective activity [39,41,42,79]. Mechanisms involved in AgNPs–antibiotic synergy have not been extensively studied, but some authors have correlated this phenomenon with the existence of an interaction between both components, which generates complexes that interact better with the bacterial envelopes and produce a higher release of Ag^+^ from the AgNPs [80], but the possibility of other mechanisms involving the proprietary mechanisms of each of the agents should not be ignored.

### 2.7. Reactive Oxygen Species (ROS) Production

**Figure 8:**
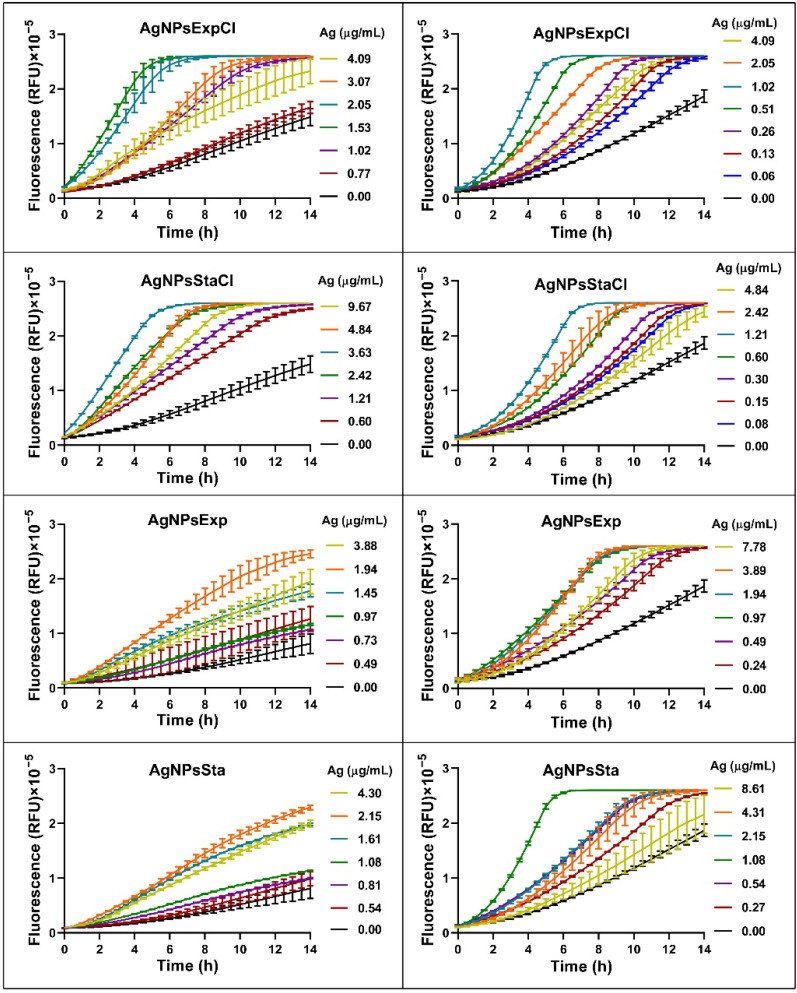
Kinetics of ROS production over time by cultures of *E. coli* ATCC 25922 (**left panels**) and *S. aureus* CECT 794 (**right panels**) in the presence of AgNPs. RFU: relative fluorescence units.

**Figure 9:**
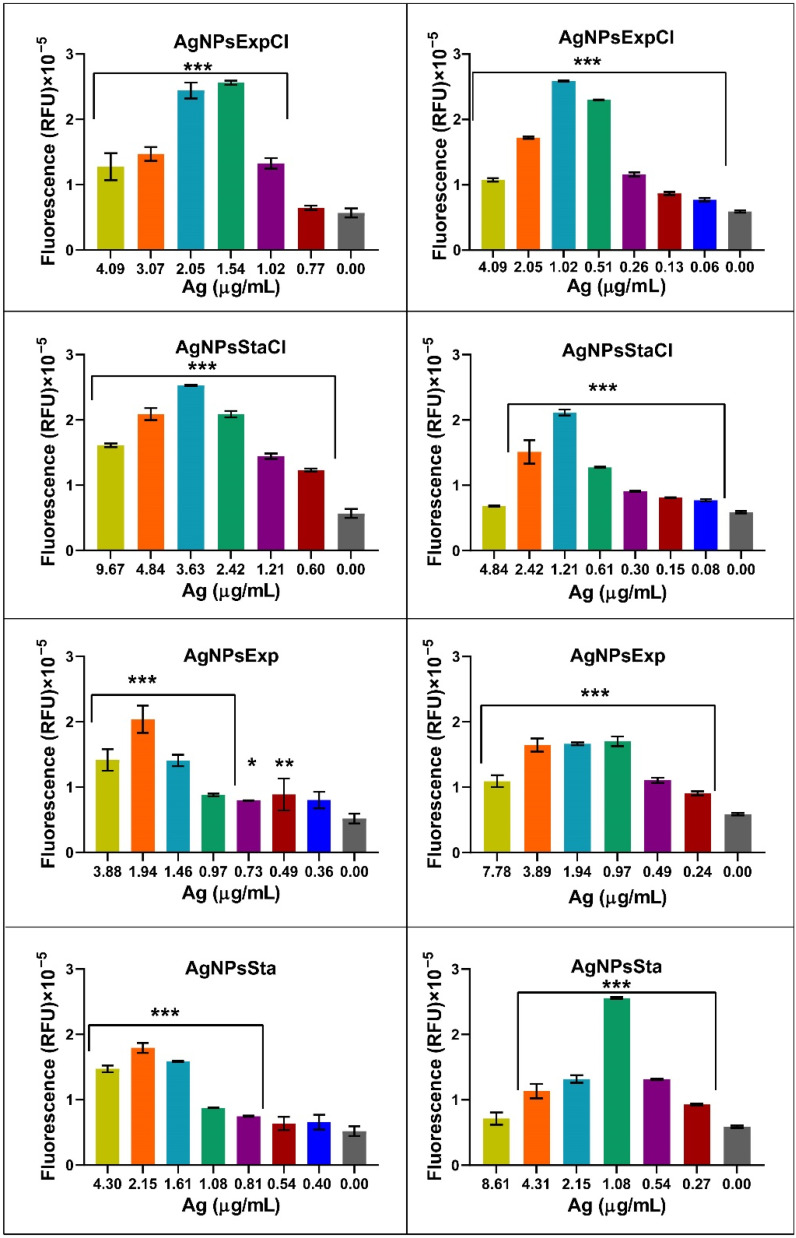
Production of ROS by cultures of *E. coli* ATCC 25922 (**left panels**) and *S. aureus* CECT 794 (**right panels**) after 6 h of incubation with AgNPs. RFU: relative fluorescence units. *p*-value: <0.05 (*), <0.01 (**), and <0.001 (***).

The production of reactive oxygen species (ROS) is generally considered one of the main mechanisms for AgNPs’ antibacterial activity. However, this is controversial and Krce et al. (2020) [81] have claimed that it is not the case, and rather that the effect on the membrane’s permeability is the main mechanism of antibacterial activity. However, the possibility still exists of ROS being one of the factors leading to the antibacterial activity of AgNPs. We have carried out a preliminary study on the production of ROS by the method generally used: measurement of fluorescence generated by the ROS effect on the structure of DCFH-DA, following an increase in fluorescence over time for several hours. The fluorescence increased over time for each AgNP in a dose-dependent way (Figure 8). For the analysis of ROS production by different nanoparticles, depending on the bacterial species used and AgNP concentrations, fluorescence produced after 6 h of incubation for each tested AgNP concentration was plotted on a graph (Figure 9). Fluorescence signals were not saturated at that time. A decrease in fluorescence was detected at the higher AgNP concentrations in all cases. This effect may be due to a methodological problem consisting of fluorescence shielding by the increased AgNP concentrations, as claimed in [81], or perhaps to other as yet unknown reasons. When considering that the AgNP concentrations producing the maximum fluorescence, differences were observed that may be related to their antibacterial activity parameters. The higher concentrations corresponded to the AgNPsStaCl, which also showed the highest MIC for *E. coli*. For *S. aureus*, the AgNPs showing the highest MIC also needed more concentration for maximum ROS production, even though in this case it was less clear. For the other AgNPs, for which MIC values were smaller and more similar, lower concentrations were needed for maximum ROS production and the differences between them were smaller, as also seen for the antibacterial activity parameters. These results support the idea that, in the two tested bacteria, the ROS production of our AgNPs is related to the efficiency of their antibacterial activity. However, more, and more precise studies of this relationship should be undertaken. Nevertheless, such a correlation does not clarify the cause–effect mechanism of the antibacterial activity. It is possible that the ROS produced by the AgNPs damages cellular components, leading to cell envelopes’ disruption and cell death, or the AgNPs first damage the envelopes, which causes the observed ROS increase, with the consecutive cellular damage that leads to the cells’ death. These two controversial models still need to be elucidated. The use of structurally related AgNPs with different antibacterial activities might help us to understand the involvement of ROS production, and other possible factors, such as envelope damage, in the antibacterial activity of AgNPs, in experiments that we would like to perform soon.

This work focused on determining the antibacterial properties of the synthesized AgNPs, but many other applications for these nanomaterials may be possible, such as biomedical ones related to antiviral, antifungal and anticancer activities as well as biosensing and photo-thermal therapies, and in other fields such as optoelectronics, bioremediation, etc. [8], which may be explored in the future.

## 3. Materials and Methods

### 3.1. Microorganisms and Culture Media

The bacterium used to prepare AgNPs was previously isolated from the Tinto River estuary (Huelva, Spain) [43]. Phylogenetic ascription of the isolate was performed based on sequence analyses of the 16S rRNA and *rpo*D genes as follows.

Genomic DNA was extracted from cells obtained by culture on nutritive agar medium and using the protocol of the Ultraclean Microbial DNA Isolation Kit (MOBIO laboratories Inc., Carlsbad, CA, USA). From the DNA obtained, PCR amplifications of 16S rDNA (primers 27F and 1492R [82] and *rpo*D (primers PsEG30F and PsEG790R [44]) were carried out in an Applied Biosystems 2720 (Applied Biosystems, Waltham, MA, USA) thermocycler. PCR reactions, in a final volume of 50 μL, contained template DNA, (1X) PCR buffer, 3 mM MgCl_2_, 0.25 μM (each) dNTPs, 0.50 μM (each) primers, and 2.5 U Taq polymerase (NZYTech, Portugal). After an initial denaturation step at 94 °C for 5 min, 30 cycles of 94 °C for 1 min, 54 °C for 1 min, and 72 °C for 1.5 min were performed, and the reaction was finished with a 10-min extension at 72 °C. For the *rpo*D amplification primers, annealing was performed at 52 °C. Amplicons were visualized by electrophoresis in a 1% agarose gel, to guarantee the quality of the DNA, and purified by the polyethylene glycol–NaCl method [83]. Finally, the DNA samples were sequenced by Macrogen (Amsterdam, The Netherlands). The GenBank accession numbers are OP422521 (16S rRNA) and OP434430 (*rpo*D). Sequences were analyzed using FinchTV 1.4.0 (https://digitalworldbiology.com/FinchTV (accessed 2 November 2022)) for sequencing chromatograms, clustal X2 (http://www.clustal.org/clustal2/ (accessed 2 November 2022) for sequence alignment and neighbor-joining tree building, and njplot (https://njplot.software.informer.com/download/ (accessed 2 November 2022)) for visualizing the phylogenetic tree. Sequences from 16S rRNA of type strains of *Pseudomonas* species used in the phylogenetic analysis were obtained from the RDP Release 11 database (http://rdp.cme.msu.edu/ (accessed 2 November 2022)). Sequences of *rpo*D genes were obtained from GenBank.

Bacterial strains used for antibacterial activity testing were the Gram-negative bacteria *E. coli* ATCC 25922, *K. pneumoniae* ATCC 2966 and *P. aeruginosa* CECT 108, PA01, and PA14, and the Gram-positive ones *B. subtilis* 168, *S. aureus* CECT 794, and *S. epidermidis* ATCC 12228.

Culture media used were nutritive broth (3 g/L meat extract (Merck Millipore, Darmstadt, Germany), 5 g/L bacteriological peptone (Condalab, Torrejón de Ardoz, Spain), 5 g/L NaCl (Merck), nutritive agar (additional 15 g/L European bacteriological agar (Condalab)), and NaCl-deprived nutritive broth. Cultures were performed at 30 °C to grow *P. alloputida* and at 37 °C to grow the test bacteria.

### 3.2. Cell-Free Broth Preparation

*P. alloputida* cultures were set in 1-L Erlenmeyer flasks containing 400 mL of medium, with or without NaCl, in a Gyrotory^®^ Water Bath Shaker model G76 (New Brunswick Scientific Co., Inc., Edison, NJ, USA) at 30 °C with shaking at 150 rpm. Exponential and stationary growth phases were sampled from both cultures. Cell-free broths were prepared by centrifugation in a Sorvall RC-5 Superspeed Floor Model Centrifuge (DuPont Instruments, Wilmington, DE, USA) at 22,200× *g* during 30 min and filtration of the supernatant through 0.22-μm pore size Millex^®^ filters (Merck-Millipore, Cork, Ireland). Samples were kept at −20 °C until use.

### 3.3. Biosynthesis of AgNPs

For AgNP biosynthesis, a 20 mM AgNO_3_ (Merck) solution in Milli-Q^®^ water, obtained with a Simplicity^®^ model of water purification system (Merck-Millipore) was added to the cell-free broths up to a 1 mM AgNO_3_ final concentration. The reaction mixture was kept at 22 °C under fluorescent light tubes (three Sylvania 18 W tubes 15 cm above the sample). Depending on the growth phase and the broth used, nanoparticles were identified as AgNPsExpCl and AgNPsStaCl, for exponential and stationary phases, respectively, of regular nutritive medium cultures (containing NaCl), and AgNPsExp and AgNPsSta, for exponential and stationary phases, respectively, for NaCl-deprived nutritive medium cultures. Color changes of the mixtures to yellow-brown indicated nanoparticle production. UV-Vis spectra (300–700 nm) of the mix were used for spectrophotometric characterization of the AgNPs over time during their synthesis for kinetics determination. After reaching maximum absorbance without aggregation, the AgNPs were purified from the rest of the mix components by centrifugation in an Eppendorf 5430 model centrifuge (Eppendorf, Hamburg, Germany) at 20,800× *g* four times, first for 15 min, and then three times for 30 min each. Afterwards, the collected pellets were mixed and washed four times with Milli-Q water, recovering AgNPs by centrifugation in the same conditions. The final pellets were resuspended in Milli-Q water at a tenth of the initial reaction volume and kept at 4 °C, for not more than one week, until use.

### 3.4. Characterization of Biosynthesized AgNPs

#### 3.4.1. UV-Visible Spectrophotometry

Formation of AgNPs, kinetics of synthesis, and final spectra of the purified AgNPs were followed by UV-Vis spectroscopy from 300–700 nm at 1 nm resolution using a microtiter plate incubator/reader model FLUOStar^®^ Omega (BMG Labtech, Offenburg, Germany).

#### 3.4.2. Transmission Electron Microscopy (TEM) of AgNPs

The shape and size of the AgNPs were determined by transmission electron microscopy (TEM) at the Centro de Biología Molecular Severo Ochoa (Madrid, Spain) using a JEM1400 JEOL microscope (Tokio, Japan) with a Oneview (Gatan Inc.-Ametek, Berwyn, PA, USA) CCD camera with a 4 K × 4 K sensor. Five microliters of a 1/50 dilution in Milli-Q water of the AgNPs stock were deposited on collodion-covered copper grids shaded with carbon, and dried before use in a Concentrator plus/Vacufuge^®^ plus (Eppendorf)) without centrifugation. Grids were kept at room temperature and protected from light. At least 500 particles of every AgNP type were analyzed with the program ImageJ64 (https://imagej.nih.gov/ij/download.html (accessed 2 November 2022)) to determine nanoparticles’ diameters. Microsoft Excel (Microsoft, Redmond, WA, USA) was used to calculate the average size and polydispersity index (PDI) using the formula PDI = (σ/γ)^2^, where σ indicates the standard deviation of the diameter size of the nanoparticles and γ their mean [84].

#### 3.4.3. Fourier Transform Infrared Spectroscopy (FTIR) of AgNPs

Fourier Transform Infrared Spectroscopy (FTIR) was performed at the Servicio Interdepartamental de Investigación (SIdI) of the Universidad Autónoma de Madrid (UAM). The wavenumber range was 4000–500 cm^−1^ with a 0.05 × 0.05 mm slit. Spectra were taken on a Spectrum Two (Perkin-Elmer, Waltham, MA, USA) for macro analysis (for AgNPs) or a Spotlight 200 (Perkin-Elmer) (for broths), on samples deposited on KBr pellets and dried in a vacuum. When broths of the reaction mixtures after synthesis were measured, a microanalysis was performed, centered on the clear zone of the prepared pellets to prevent the interference of residual nanoparticles.

#### 3.4.4. X-ray Diffraction of AgNPs

X-ray diffraction (XRD) analysis was performed at the SIdI of the UAM, using a model X’Pert PRO theta/2theta X-ray diffractometer (Malvern Panalytical, Malvern, UK), with a primary germanium monochromator (monochromator Johansson) and an X’Celerator fast detector.

#### 3.4.5. Total Reflection X-ray Fluorescence (TXRF)

This technique was used for determining AgNPs’ composition and the silver concentration of AgNP suspensions at the SIdI of the UAM, using a S2 PicoFox TXRF spectrometer (Bruker, Ettlingen, Germany), equipped with a Mo X-ray source working at 50 kV and 600 µA, a multilayer monochromator with 80% reflectivity at 17.5 keV (Mo K), and an XFlash SDD detector with an effective area of 30 mm^2^ and an energy resolution of better than 150 eV for 5.9 keV (Mn Kα). For deconvolution and integration, the commercial software package Spectra v. 7.5.3 (Bruker) was used.

#### 3.4.6. Zeta Potential and Hydrodynamic Diameter of AgNPs

These parameters were measured by dynamic light scattering (DLS) using a Zetasizer Ultra (Malvern Panalytical, Malvern, UK), at the Dept. of Material Physics of the UAM, using samples diluted in Milli-Q water.

### 3.5. Antibacterial Activity of AgNPs

#### 3.5.1. Microdilution Method

Cultures in nutritive medium were set in 96-well microplates (Sarstedt, Nümbrecht, Germany), with a final volume of 200 µL per well. The test bacteria were adjusted to 5 × 10^5^ CFU/mL, and 1/2 serial dilutions of the stock suspensions of the AgNPs were distributed in the wells. The corresponding negative controls were also set. Microplates were incubated overnight at 37 °C, in the same microtiter plate incubator/reader indicated above. Absorbance at 660 nm was registered every hour, after 20 s shaking. All samples were implemented at least in triplicate. MIC was determined as the AgNPs concentration in the well with no apparent growth. For MBC determination, 50-μL aliquots of cultures in the wells corresponding to the MIC and two concentrations immediately above were plated in nutritive agar in duplicate. After overnight incubation at 37 °C, the MBC was calculated after colony counting and determination of the concentration of AgNPs corresponding to a killing of at least 99.9% of the original viable bacteria. IC_50_ values were calculated using GraphPad Prism VIII (GraphPad software, San Diego, CA, USA) from the growth inhibition data corresponding to the incubation times in which the growth controls reached the early stationary phase.

#### 3.5.2. Antibiofilm Assay

Biofilm formation was evaluated by a crystal violet assay [85] with some modifications. The broths in the microplates, used for growth inhibition activity determination, were replaced with 200 μL of methanol (Honeywell, Seelze, Germany) and the microplates incubated for 20 min at RT to fix biofilms. Subsequently, methanol was replaced with 200 μL of a crystal violet (Sigma-Aldrich, St. Louis, MO, USA) 0.5% solution in Milli-Q water and the plates incubated for 20 min. After removing the colorant solution, the biofilm was washed four times with 300 μL of distilled water to remove the excess dye and dried at RT. To dissolve the dye that adhered to the biofilm, 200 μL of 96% (*v*/*v*) ethanol (VWR^®^ Chemicals, Ronsy-sous-Bois, France) were added. Measurement of the dissolved crystal violet was performed at 590 nm after 30 min and after 24 h of incubation. The highest of the two readings was considered.

### 3.6. Synergy of AgNPs with Classic Antibiotics in Growth Inhibition

A checkerboard type of assay [76] was used. Test bacteria for these assays were *E. coli* ATCC 25922 and *S. aureus* CECT 794. Three classical antibiotics with different cellular targets were chosen: ampicillin, nalidixic acid, and streptomycin. The assays were carried out in 96-well microplates. The MIC of each antibiotic for each microorganism was determined previously by microdilution. Half dilutions of each antibiotic and AgNPs were prepared and distributed throughout the plate. In each well, 90 μL of bacterial culture at 1 × 10^6^ CFU/mL were added, together with 90 μL of fresh nutritive broth, 10 μL of AgNP dilutions, and 10 μL of the corresponding antibiotic diluted solutions. Negative controls, and AgNPs and antibiotics alone, were included in the microplates. Experiments were performed in duplicate. Microplates were incubated at 37 °C for 24 h. The MIC of each antibiotic and AgNP were confirmed in the same plate as their combinations. The fractional inhibitory concentration index (FICI) [75,76] was calculated, as well as the modulatory factor (MF) [40].

### 3.7. Reactive Oxygen Species (ROS) Detection

ROS production tests were carried out with 2′-7′-dichlorodihydrofluorescein diacetate (DCFH-DA) (Sigma-Aldrich), based on the protocols described [81,86], using *E. coli* ATCC 25922 and *S. aureus* CECT 794. Bacterial cultures in the exponential phase were pelleted at 9400× *g* and resuspended in a PBS buffer. Bacteria at 7.5 × 10^7^ CFU/mL and DCFH-DA at a 5 μM final concentration were mixed in 96-well microplates. To the 190 μL of mixture, 10 μL of AgNPs at different concentrations, above and below their MICs, were added. Milli-Q water was used as a negative control and 0.33 mM H_2_O_2_ as a positive one. Plates were incubated aerobically at 37 °C for 14 h, measuring every half hour, with prior agitation at 300 rpm, using an excitation of 485–12 nm and an emission of 520 nm. The fluorescence produced after 6 h of incubation was used to compare ROS production in the presence of the different AgNPs.

### 3.8. Statistical Analysis

The values of IC_50_ and ICb_50_ against each bacterium were analyzed using a two-tailed unpaired *t*-test with a *p*-value of 0.05. Values for AgNPs were tested to determine differences between those prepared with the same type of broth, presence or not of NaCl, and growth phase origin. The results of ROS detection were analyzed using a two-way ANOVA and Dunnett’s multiple comparison, with a *p*-value = 0.05 to detect significant differences between the negative control and the presence of different concentrations of AgNPs.

## 4. Conclusions

In this work, four types of AgNPs were produced by a green method, based on the use of broths from exponential and stationary growth phases of *P. alloputida* cultures in media with or without NaCl, reusing the broths for three successive batches. These AgNPs were shown to be good metal-based antibacterial drugs, especially against *P. aeruginosa* strains. These AgNPs can perform synergistically with classic antibiotics, decreasing the concentrations needed of both agents in the inhibition of bacterial growth, which may be of interest for fighting infections and lowering toxicity against the bacterial hosts. We have also shown that the ROS production promoted by these nanomaterials is related to their antibacterial activity, suggesting that they can be useful tools for studying the mechanisms involved in the antibacterial activity of Ag-based drugs.

## Figures and Tables

**Table 1:** Summary of the AgNPs’ characterization.

(AgNPs)	Z-Potential (mV)	Diameter (nm) (DLS)	PDI (DLS)	Diameter (nm) (TEM)	PDI (TEM)
AgNPsExpCl	−35.260 ± 3.295	41.760 ± 0.392	0.305 ± 0.001	9.290 ± 4.530	0.238
AgNPsStaCl	−19.850 ± 0.523	48.900 ± 1.746	0.343 ± 0.051	19.160 ± 9.450	0.243
AgNPsExp	−30.980 ± 5.893	28.780 ± 0.149	0.455 ± 0.001	8.301 ± 3.777	0.207
AgNPsSta	−23.500 ± 2.898	62.750 ± 0.918	0.304 ± 0.001	7.336 ± 5.875	0.415

**Table 2:** Antimicrobial and antibiofilm activity.

Test Bacteria	AgNPs	MIC (μg/mL)	MBC (μg/mL)	IC_50_ (μg/mL)	ICb_50_ (μg/mL)
*E. coli* ATCC 25922	AgNPsExpCl	1.01	1.34	0.12 ± 0.07 **	0.40 ± 0.05 **/##
	AgNPsStaCl	4.84	4.84	1.46 ± 0.34 **/#	2.75 ± 0.84 **/#
	AgNPsExp	0.49	0.49	0.02 ± 0.00 ***	0.14 ± 0.01 ***/##
	AgNPsSta	1.08	1.08	0.57 ± 0.05 ***/#	0.67 ± 0.03 ***/#
*K. pneumoniae* ATCC 29665	AgNPsExpCl	2.01	2.01	0.57 ± 0.16 */##	1.02 ± 0.91 *
	AgNPsStaCl	9.68	9.68	1.85 ± 0.73 */#	3.65 ± 0.54 */##
	AgNPsExp	0.97	0.97	0.06 ± 0.03 */##	0.54 ± 0.10 **
	AgNPsSta	1.08	1.08	0.48 ± 0.19 */#	1.13 ± 0.16 **/##
*P. aeruginosa* CECT 108	AgNPsExpCl	1.01	2.01	0.09 ± 0.04 */#	0.55 ± 0.03 ***/###
	AgNPsStaCl	2.42	4.84	0.55 ± 0.24 */#	1.31 ± 0.09 ***/###
	AgNPsExp	0.24	0.97	0.01 ± 0.00 **/#	0.13 ± 0.01 ***/###
	AgNPsSta	0.54	1.08	0.16 ± 0.02 **/#	0.35 ± 0.03 ***/###
*P. aeruginosa* PA01	AgNPsExpCl	-	-	-	-
	AgNPsStaCl	-	-	-	-
	AgNPsExp	-	-	-	-
	AgNPsSta	0.54	1.08	0.09 ± 0.01	0.32 ± 0.05
*P. aeruginosa* PA14	AgNPsExpCl	-	-	-	-
	AgNPsStaCl	-	-	-	-
	AgNPsExp	-	-	-	-
	AgNPsSta	0.54	1.08	0.08 ± 0.04	0.25 ± 0.03
*S. aureus* CECT 794	AgNPsExpCl	4.03	4.03	0.58 ± 0.15 **/##	0.60 ± 0.34 ***
	AgNPsStaCl	9.68	9.68	4.64 ± 1.05 **/##	6.19 ± 0.08 ***/###
	AgNPsExp	1.94	3.89	0.06 ± 0.01 ***/##	1.05 ± 0.43
	AgNPsSta	1.44	2.15	0.82 ± 0.15 ***/##	1.14 ± 0.10 ###
*S. epidermidis* ATCC 12228	AgNPsExpCl	0.51	4.03	0.25 ± 0.05 ***/##	0.40 ± 0.24 **
	AgNPsStaCl	4.84	9.68	0.72 ± 0.02 ***/###	1.90 ± 0.25 **/###
	AgNPsExp	0.49	1.94	0.01 ± 0.00 **/##	0.23 ± 0.09 *
	AgNPsSta	1.08	4.31	0.29 ± 0.06 **/###	0.54 ± 0.09 */###
*B. subtilis* 168	AgNPsExpCl	1.01	1.01	0.40 ± 0.08 **/##	0.24 ± 0.07 ***/#
	AgNPsStaCl	3.23	3.23	2.47 ± 0.65 **/##	0.70 ± 0.09 **/#
	AgNPsExp	0.48	0.49	0.02 ± 0.01 **/##	0.10 ± 0.03 #
	AgNPsSta	1.08	1.08	0.53 ± 0.15 **/##	0.50 ± 0.08 **/#

Antibacterial activity parameters are indicated as Ag concentrations; * indicates significant differences between AgNPs prepared from the same medium but different growth phase; # indicates significant differences between AgNPs from the same growth phase but from different media. *p*-value: <0.05 (*) or (#); <0.01 (**) or (##); <0.001 (***) or (###).

**Table 3:** Synergy effects of the AgNPs in combination with classic antibiotics against *E. coli* and *S. aureus*.

(AgNPs)	Test Bacteria	Ampicillin		Nalidixic Acid		Streptomycin	
		FICI	MF	FICI	MF	FICI	MF
AgNPsExpCl	*E. coli* ATCC 25922	2.000	1	0.750	2	0.047	32
	*S. aureus* CECT 794	0.500	2	2.000	1	0.047	16
AgNPsStaCl	*E. coli* ATCC 25922	2.000	1	1.000	1	0.180	32
	*S. aureus* CECT 794	0.375	4	2.000	1	0.125	16
AgNPsExp	*E. coli* ATCC 25922	2.000	1	0.625	2	0.094	32
	*S. aureus* CECT 794	0.375	4	2.000	1	0.078	16
AgNPsSta	*E. coli* ATCC 25922	2.000	1	2.000	1	0.039	32
	*S. aureus* CECT 794	0.375	4	2.000	1	0.039	32

## Data Availability

Not applicable.

## Supplementary Materials

The following supporting information can be downloaded at https://www.mdpi.com/article/10.3390/molecules27217589/s1: Figure S1: Growth curves of *Pseudomonas alloputida* B003 UAM on regular and NaCl-deprived nutritive media. Figure S2: Stability of UV-Vis spectra of AgNPs before and after washing. Figure S3: Stability of AgNPsStaCl and AgNPsSta at 4 °C. Figure S4: Conservation of MIC and MBC values of AgNPsStaCl (left) and AgNPsSta (right). Figure S5: Reuse of broths for synthesis of AgNPsStaCl.

## Sample Availability

Samples of *P. alloputida* B003 UAM are available from the authors.
