# Supplementary figures and images for: Green Extracellular Synthesis of Silver Nanoparticles by Pseudomonas alloputida, Their Growth and Biofilm-Formation Inhibitory Activities and Synergic Behavior with Three Classical Antibiotics
Source: Molecules. 2022 Nov 5;27(21):7589. doi: 10.3390/molecules27217589 (PMC9656067; doi:10.3390/molecules27217589)

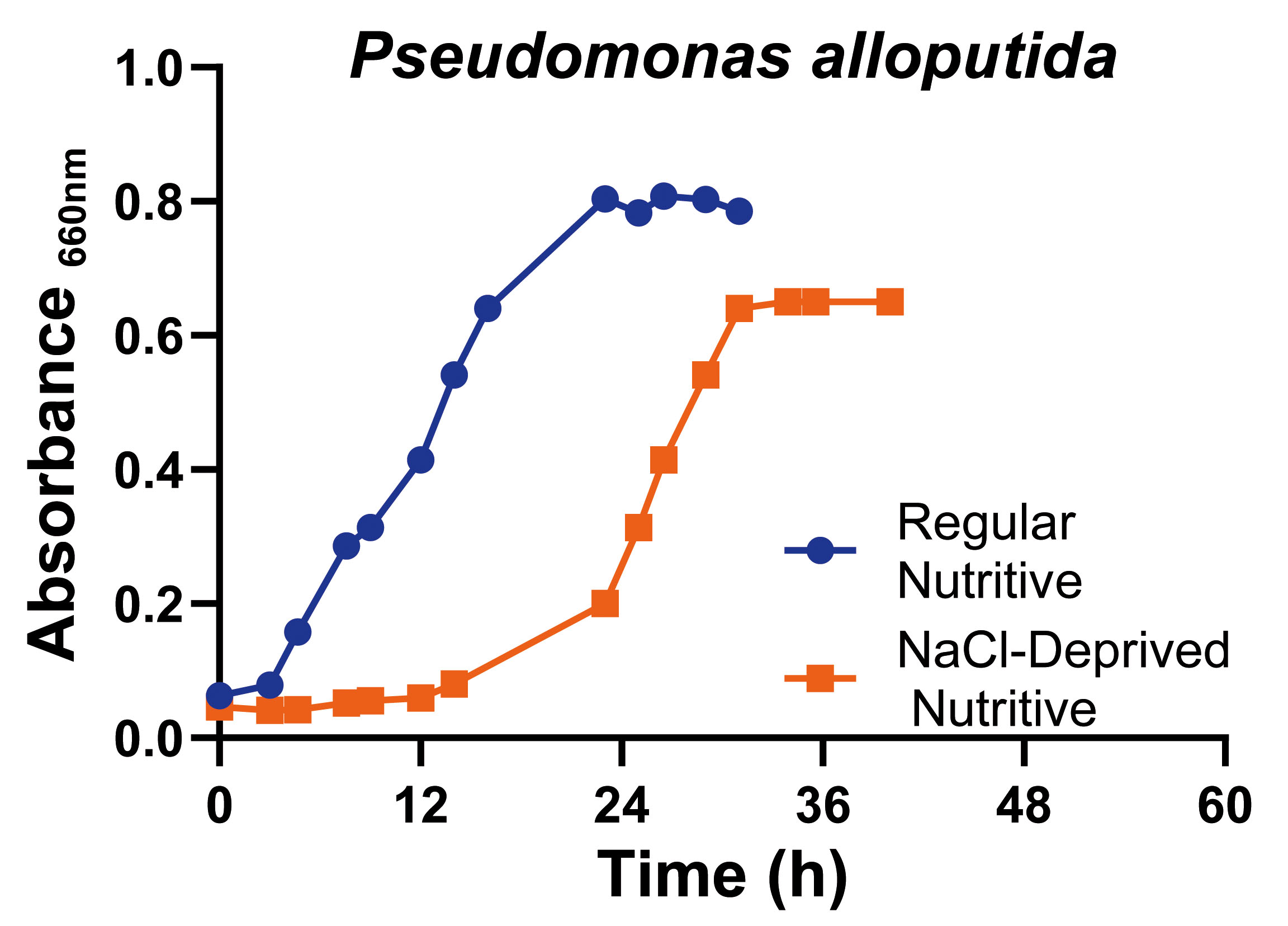

Supplement: Supplementary file 1 [file molecules-27-07589-s001.zip › Suplementary figures/Figure-S1.jpg]

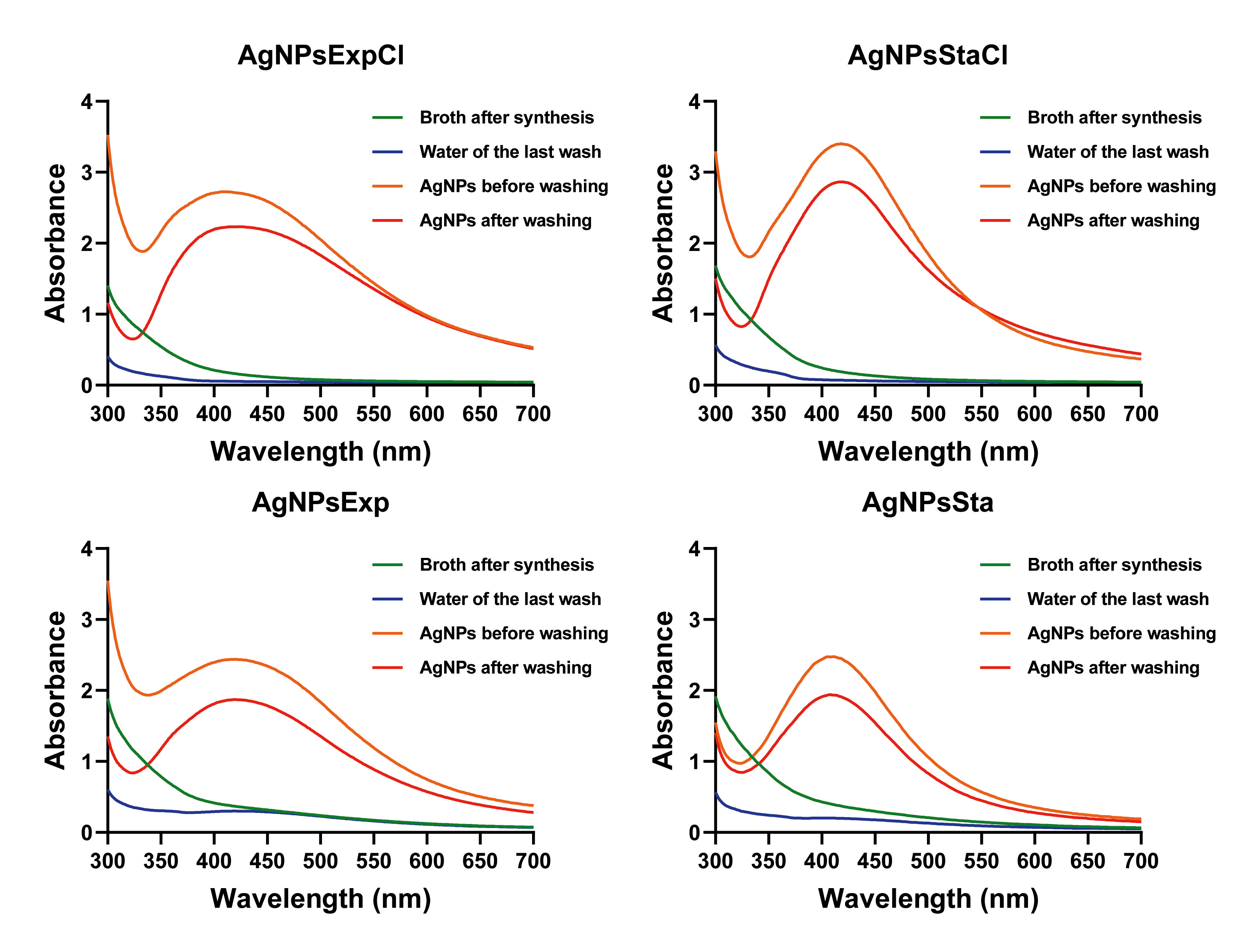

Supplement: Supplementary file 1 [file molecules-27-07589-s001.zip › Suplementary figures/Figure-S2.jpg]

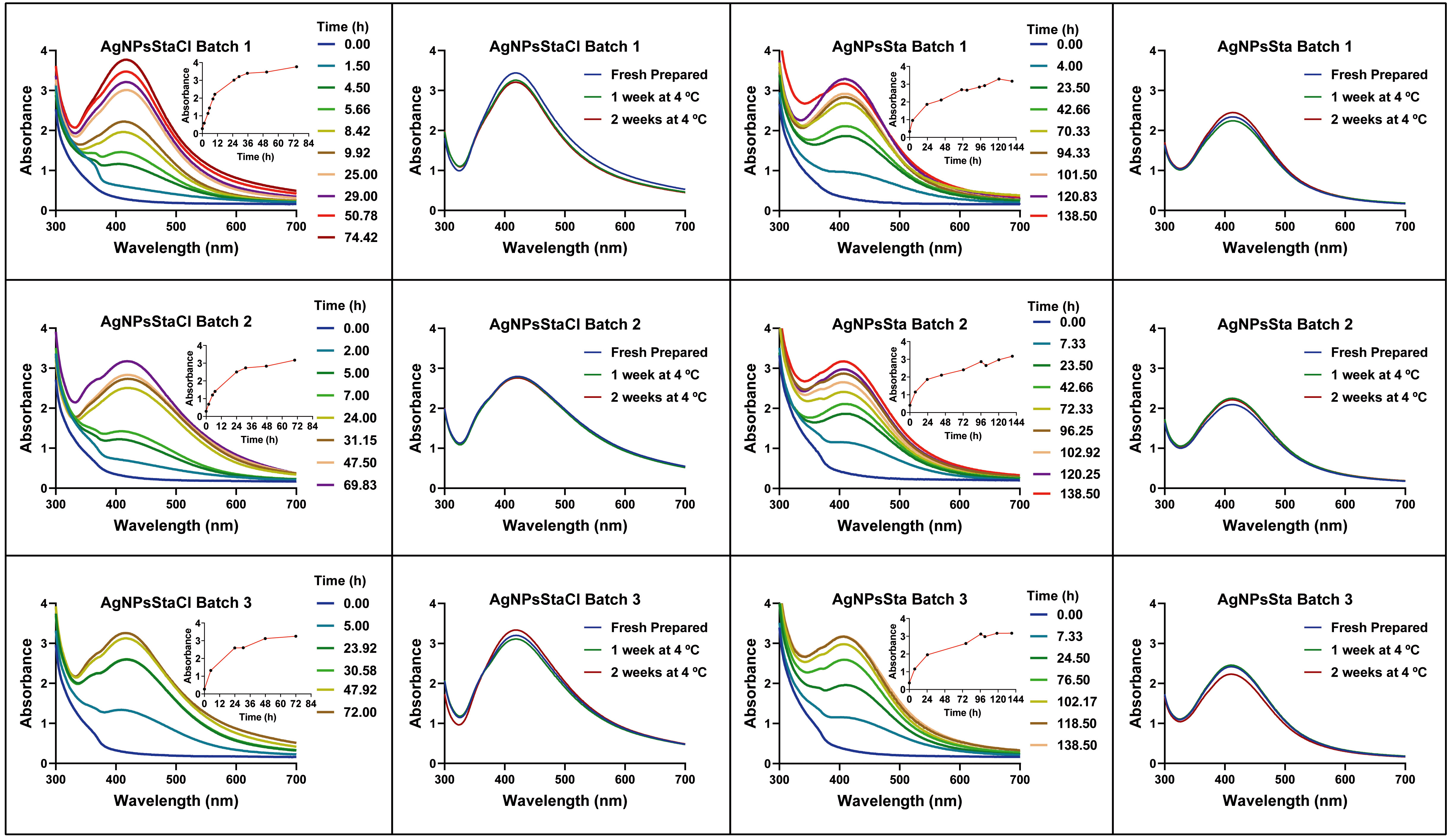

Supplement: Supplementary file 1 [file molecules-27-07589-s001.zip › Suplementary figures/Figure-S3.jpg]

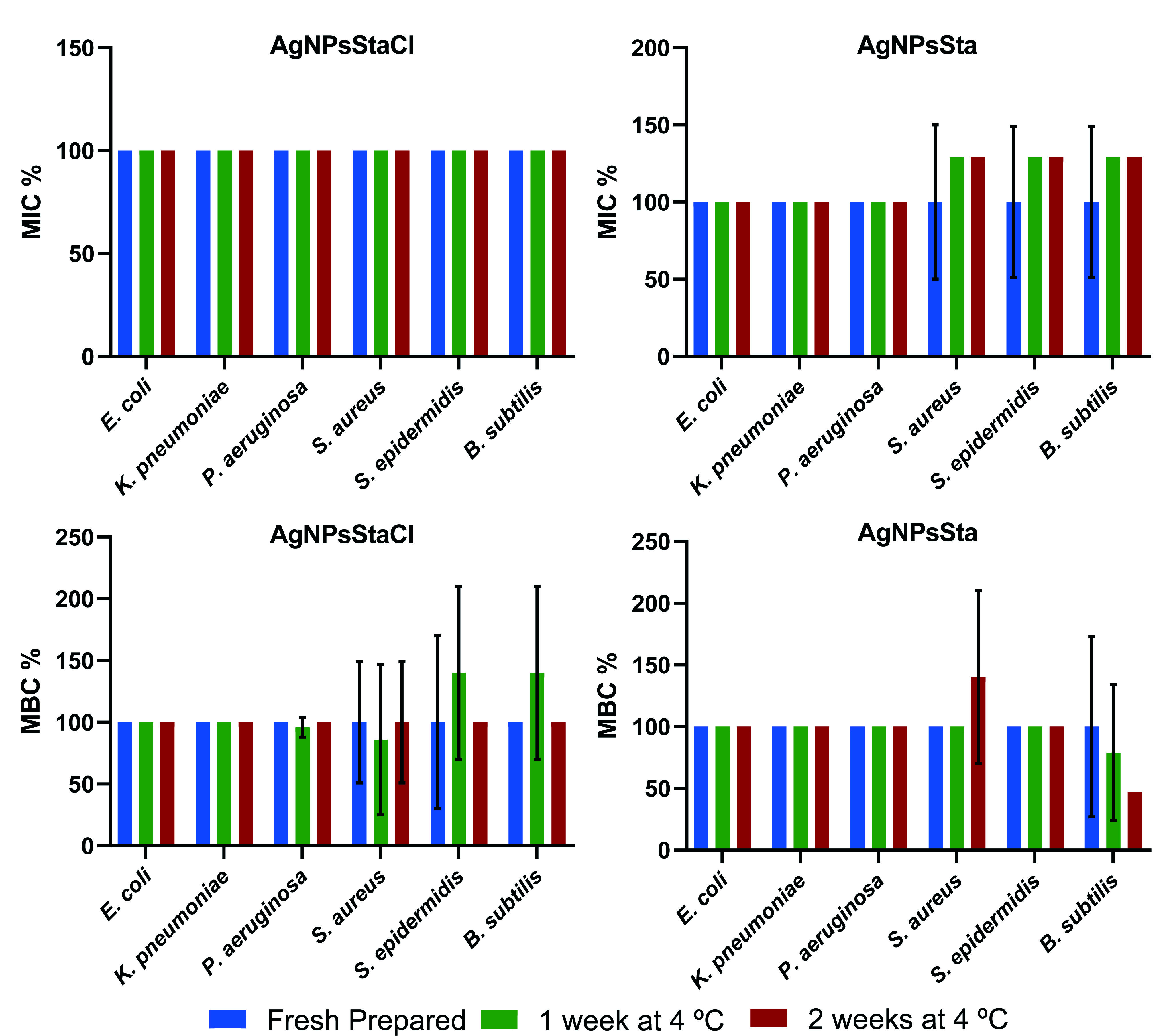

Supplement: Supplementary file 1 [file molecules-27-07589-s001.zip › Suplementary figures/Figure-S4.jpg]

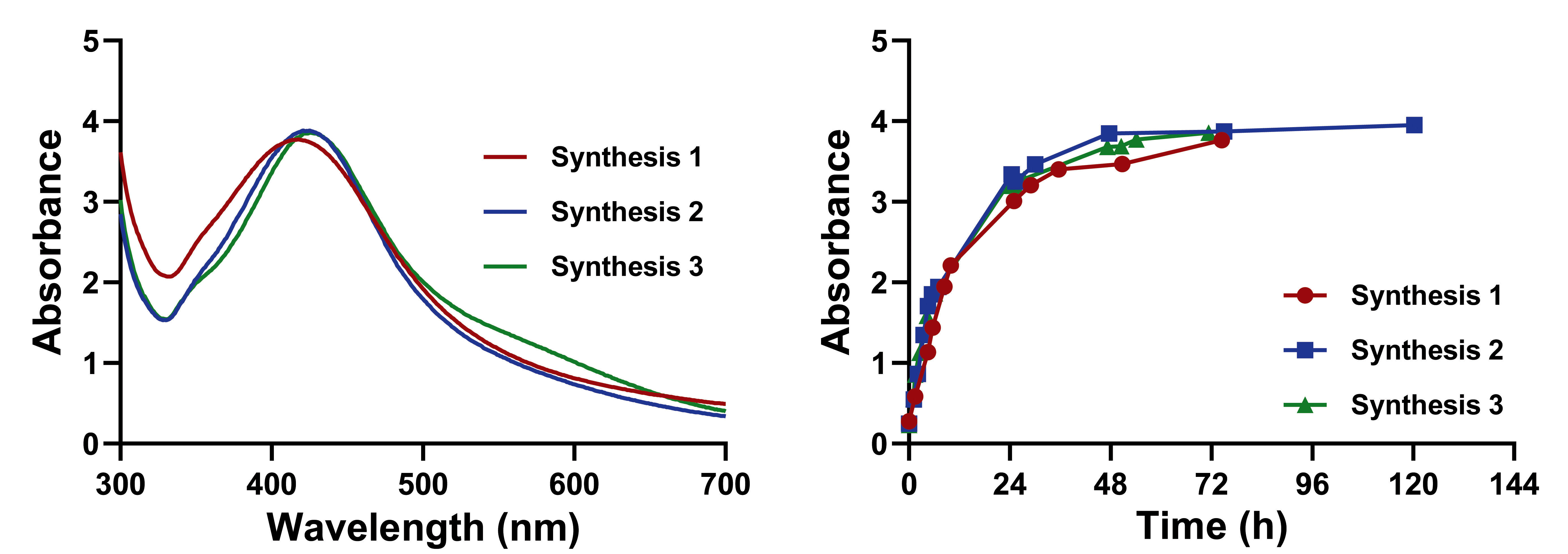

Supplement: Supplementary file 1 [file molecules-27-07589-s001.zip › Suplementary figures/Figure-S5.jpg]
